# HDL-S1P: cardiovascular functions, disease-associated alterations, and therapeutic applications

**DOI:** 10.3389/fphar.2015.00243

**Published:** 2015-10-20

**Authors:** Bodo Levkau

**Affiliations:** Institute for Pathophysiology, West German Heart and Vascular Center, University Hospital Essen, Essen, Germany

**Keywords:** sphingosine-1-phosphate, sphingolipids, High-density lipoproteins, coronary artery disease, HDL dysfunction, HDL-S1P

## Abstract

Sphingosine-1-phosphate (S1P) is a bioactive sphingolipid contained in High-density lipoproteins (HDL) and has drawn considerable attention in the lipoprotein field as numerous studies have demonstrated its contribution to several functions inherent to HDL. Some of them are partly and some entirely due to the S1P contained in HDL (HDL-S1P). Despite the presence of over 1000 different lipids in HDL, S1P stands out as it possesses its own cell surface receptors through which it exercises key physiological functions. Most of the S1P in human plasma is associated with HDL, and the amount of HDL-S1P influences the quality and quantity of HDL-dependent functions. The main binding partner of S1P in HDL is apolipoprotein M but others may also exist particularly under conditions of acute S1P elevations. HDL not only exercise functions through their S1P content but have also an impact on genuine S1P signaling by influencing S1P bioactivity and receptor presentation. HDL-S1P content is altered in human diseases such as atherosclerosis, coronary artery disease, myocardial infarction, renal insufficiency and diabetes mellitus. Low HDL-S1P has also been linked to impaired HDL functions associated with these disorders. Although the pathophysiological and molecular reasons for such disease-associated shifts in HDL-S1P are little understood, there have been successful approaches to circumvent their adverse implications by pharmacologically increasing HDL-S1P as means to improve HDL function. This mini-review will cover the current understanding of the contribution of HDL-S1P to physiological HDL function, its alteration in disease and ways for its restoration to correct HDL dysfunction.

Plasma levels of High density lipoprotein cholesterol (HDL-C) are the best known negative predictors of clinical atherosclerosis. Besides their crucial role in reverse cholesterol transport (RCT) HDL exercise numerous biological functions that are independent of RCT and potentially contributory to their beneficial effects. Sphingosine-1-phosphate (S1P) is among several of the molecules carried by HDL that have been implicated as biochemical correlates of these functions particularly because both alterations of HDL-S1P content and interference with genuine S1P signaling substantially them. This mini-review focuses on the current understanding of how HDL-S1P contributes to such HDL functions and its relevance as a disease marker and possibly therapeutic target in human disorders characterized by dysfunctional HDL.

## Physiology of HDL-S1P: Sources, Uptake, and Metabolism

The S1P amount in human plasma ranges between 200 and 1000 nM and the majority of it (65–80%) is associated with the HDL fraction (Okajima, 2002; Zhang et al., 2005; Sattler et al., 2010) The remainder is contained in other lipoproteins and associated with serum albumin, respectively. The amphipathic S1P molecule needs binding to such carriers to be present in biological fluids. The main plasma apolipoprotein to which S1P physically binds to is apolipoprotein M (apoM; Christoffersen et al., 2011), the vast majority of which is contained in HDL (Arkensteijn et al., 2013). In fact, the presence of apoM determines HDL-S1P: only the apoM-containing fraction of human HDL carries S1P, and apoM-deficient mouse HDL contains almost no S1P (Christoffersen et al., 2011). However, there is no correlation of apoM and S1P in human plasma (Karuna et al., 2011) or HDL (own data). On a stoichiometric basis, one in 10 HDL particles contains S1P (Poti et al., 2014) but only one in 20 contains apoM (Arkensteijn et al., 2013). In addition, the lipophilic pocket of apoM that accommodates S1P also serves as a binding domain for other molecules such as retinol and oxidized phospholipids that may compete with S1P for binding (Arkensteijn et al., 2013). This suggests that there may be other molecular partners for S1P within HDL besides apoM and that their presence and/or association with S1P may depend on the equilibrium with apoM-associated S1P.

Early studies using lipid-depleted charcoal-treated plasma have demonstrated in cell-based assays that only 2% of the plasma S1P is biologically active (Murata et al., 2000). This may explain why luminal endothelial S1P receptors that are constantly exposed to plasma S1P concentrations ∼20–100-fold higher than their K_*d*_ values (Mandala et al., 2002) are not desensitized but, instead, effectively respond to administration of S1P as well as agonists and antagonists of its receptors. At the same time, this also suggests that either only a small part of the S1P contained in HDL is biologically active or that just part of the HDL-bound pool of plasma S1P has simultaneous access to S1P receptors. While the pursuit of the first notion requires clarification of the exact structural topology and dynamics of S1P as well as its movements within HDL, the second would be conceptually fulfilled if S1P required a handoff by a genuine HDL receptor in order to either engage an adjacent S1P receptor or translocate to the membrane and glide successively into the binding pocket of the S1P receptor. There is support in the literature in favor of the scavenger receptor BI (SR-BI), a genuine HDL receptor, providing such a handoff particularly because of the considerable overlap between S1P-dependent and SR-BI-mediated functions of HDL (Yuhanna et al., 2001; Nofer et al., 2004; Kimura et al., 2006; Tolle et al., 2008).

The physiological sources from which HDL receive their S1P content are currently unknown. They may be the same as those giving rise to the overall plasma S1P. As plasma constituents with the highest affinity for S1P, HDL may represent the primary binding destination for S1P and/or extract S1P from low-affinity carriers. This is feasible as HDL are in permanent physical and metabolic contact with other plasma components including a variety of lipoproteins which would allow such S1P transfer to take place. In support of this scenario, the phospholipid transfer protein, a plasma protein that mediates the exchange of cholesterol, phospholipids and other amphiphilic molecules among lipoproteins, has been demonstrated to be involved in maintaining the S1P content of HDL (Yu et al., 2013). A second option would be HDL acquiring S1P directly from cells and organs that they are in constant contact with. Indeed, HDL can take up S1P directly from erythrocytes and do so through physical contact with the plasma membrane (Bode et al., 2010; Sattler et al., 2015). Whether this constitutes a passive diffusion process or involves the biochemically characterized but yet unidentified erythrocyte ATP/ADP-dependent S1P transporter that transfers S1P to albumin (Kobayashi et al., 2009) is still unknown. Recently, a S1P-specific transporter—Spinster homolog 2 (Spns2)—has been identified as major player in S1P release and secretion predominantly in lymphatic (Mendoza et al., 2012) but also vascular endothelial cells (Fukuhara et al., 2012). Endothelial-specific Spns2-deficient mice have diminished S1P content in their HDL fraction but this is does not suggest Spns2 transporting S1P specifically to HDL as it may simply reflect the ∼50% reduction in their overall plasma S1P (Fukuhara et al., 2012).

Instead, it nicely exemplifies that general reduction of plasma S1P goes along with reduction of the S1P amount contained within the entire HDL fraction. Nevertheless, one study hints at the possible existence of HDL-specific S1P transporters at least in astrocytes where the presence/accumulation of S1P in the extracellular space was coupled to the formation of apolipoprotein E-containing HDL-like lipoproteins through the ATP-binding cassette (ABC) transporter ABCA1 (Sato et al., 2007). However, this observation may be restricted to the brain as plasma S1P is not reduced in mice deficient for ABCA1, ABCA7, or ABCC1 (Lee et al., 2007). Still the idea that S1P incorporation into lipoproteins can occur at the very early steps of nascent HDL formation where ABCA1 plays an indispensable role is appealing. Recently, it has gained support by the observation that hepatocytes—the cells instrumental for HDL generation—secrete S1P in complex with apoM, and that hepatocyte-specific apoM-transgenic mice feature S1P-rich HDL particles and an increased synthesis and release of S1P (Liu et al., 2013). While the first observation may be a function of more apoM being targeted to HDL as their predominant destination and extracting S1P on its way there, the second is a hint that apoM may be, indeed, a genuine S1P carter hauling it across the hepatocyte membrane and integrating it in complex with itself into HDL. These observations suggest that S1P incorporation into HDL may be initiated very early during the HDL generation process and proceeds throughout HDL maturation.

## Established S1P-dependent HDL Functions

There are many studies that have mapped individual HDL functions as partially or sometimes completely attributable to HDL-S1P (for detailed review, see Sattler and Levkau, 2009; Sato and Okajima, 2011; Poti et al., 2014). Blocking HDL-S1P with a neutralizing antibody (Sphingomab; Sattler et al., 2015), S1P removal by delipidation (Murata et al., 2000) and the omission to substitute S1P during the manufacturing of reconstituted HDL (Brulhart-Meynet et al., 2015) all result in inferior HDL function. Clear-cut proof comes also from experiments where HDL effects are attenuated by S1P receptor antagonists or in the respective genetic knockouts or knockdowns. Some of the most prominent HDL functions shown to depend on HDL-S1P are listed here: (1) nitric oxide (NO) production by the endothelial NO synthase (eNOS; Nofer et al., 2004); (2) eNOS/NO-dependent vasodilatation in explanted arteries and *in vivo* (Nofer et al., 2004); (3) inhibition of TNF*α*-induced adhesion molecule expression in endothelial cells (Kimura et al., 2006); (5) strengthening of endothelial cell barrier (Wilkerson et al., 2012); (6) endothelial cell proliferation, survival, tube formation and angiogenesis (Kimura et al., 2003); (7) induction of long pentraxin 3 (Norata et al., 2008) and TGFβ in endothelial cells (Norata et al., 2005); (8) migration and angiogenesis promoted by endothelial lipase (Tatematsu et al., 2013); (9) protection of single cardiomyocytes and hearts *in vivo* against ischemic, hypoxic and reperfusion injury(Theilmeier et al., 2006; Frias et al., 2009a,b; Tao et al., 2010); (10) inhibition of migration (Tamama et al., 2005; Damirin et al., 2007), thrombin-induced NADP(H) oxidase activation and monocyte chemoattractant protein-1 (MCP-1) production (Tolle et al., 2008) in smooth muscle cells (SMC); (11) desensitization of guanyl cyclase B (Chrisman et al., 2003) by HDL, HDL-extracted lipids and authentic S1P, and (12) upregulation of prostacyclin production and COX2 in SMC (Gonzalez-Diez et al., 2008). However, the most crucial and physiologically relevant function of HDL—the participation in reverse cholesterol transport—is not affected by the presence of S1P within HDL, as measured in surrogate assays where reconstituted HDL with and without S1P were similarly effective in stimulating cholesterol efflux (Matsuo et al., 2007). In summary, the many S1P-attributed functions of HDL should clearly be considered when overall HDL bioactivity is being assessed.

Interestingly, there is recent evidence that S1P may exert different biological functions dependent on whether it acts in HDL-associated or albumin-associated form. One such observation has been made in cultured endothelial cells where HDL-S1P was longer effective at maintaining endothelial barrier function than albumin-S1P despite the similar half-life, which the authors contributed to a lower rate of S1P_1_ internalization and degradation by HDL-S1P than albumin-S1P (Wilkerson et al., 2012). *In vivo*, apoM-deficient mice that virtually lack HDL-S1P display vascular leakage in the lungs but their plasma S1P is also reduced by half (Christoffersen et al., 2011). As sphingosine kinase 1-deficient mice featuring a comparable reduction in plasma S1P (Allende et al., 2004) also display an increase in vascular leakiness (Li et al., 2008) there is no evidence for an exclusive role of HDL-S1P in protecting against it. Furthermore, many studies have shown that administration of genuine S1P and S1P receptor agonists protect against endothelial leakage (McVerry and Garcia, 2005; Wang and Dudek, 2009; Abbasi and Garcia, 2013; Wilkerson and Argraves, 2014). Nevertheless, a recent study reiterated the possibility that HDL-S1P may have unique functions compared to S1P presented by other carriers: HDL containing apoM-bound S1P but neither apoM-deficient HDL nor albumin-bound S1P restrained lymphopoiesis and neuroinflammation (Blaho et al., 2015) although the mechanisms have remained unclear. They may be manifold such as, e.g., differences in S1P presentation mode and the alleged assistance of HDL receptors in S1P signaling along with differences in S1P half-life between the different carrier-bound forms (the metabolism of HDL-associated S1P is fourfold slower than that of albumin-associated S1P, Kimura et al., 2001). Future work is needed to delineate the exact mechanisms and mode of action.

## Alterations of HDL-S1P in Human Cardiovascular, Kidney, and Metabolic Diseases

**FIGURE 1:**
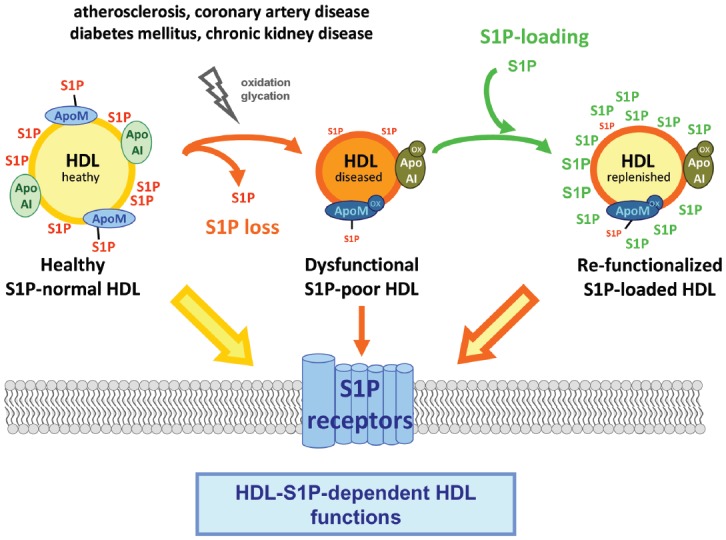
**Model of HDL-S1P functions, their impairment in disease and the therapeutic potential they bear by replenishing or increasing the S1P content of HDL**.

If the amount of S1P carried by HDL was important for physiological HDL function in men, human diseases where HDL function is known to be impaired should be examined for altered HDL-S1P. HDL dysfunction is a common feature in patients with atherosclerotic disease, diabetes mellitus, metabolic syndrome and chronic kidney disease and presents with compromised anti-oxidative, anti-inflammatory, anti-apoptotic, vasodilative and cholesterol efflux properties (Kontush and Chapman, 2006; Barter et al., 2007; deGoma et al., 2008; Besler et al., 2011). The molecular determinants of HDL dysfunction are still insufficiently understood and generally sought in the quantitative and qualitative changes occurring in the HDL proteome and lipidome including modifications such as oxidation and glycation (Vaisar et al., 2007; Kontush et al., 2013; Riwanto et al., 2013). Thus the causal relationships between individual molecular changes in HDL and their corresponding functional deficits are largely unknown. Accordingly, any HDL constituent such as S1P that is identified to participate in HDL function becomes a candidate for investigation in clinical disease settings (Figure 1).

A number of studies have linked plasma and HDL-S1P to the incidence of coronary artery disease (CAD): (1) HDL-S1P is lower in patients with stable CAD than in healthy individuals (Sattler et al., 2010; Argraves et al., 2011); (2) HDL-S1P correlates inversely with the occurrence of CAD independently of HDL-C (Argraves et al., 2011); (3) HDL-S1P correlates negatively with the severity of coronary atherosclerosis (Sattler et al., 2014), and (4) HDL-S1P is an independent predictor of coronary in-stent restenosis (Jing et al., 2015). In patients with acute myocardial infarction (AMI), both plasma and HDL-S1P levels are increased early after the onset of symptoms and decrease thereafter (Sattler et al., 2010). Transient myocardial ischemia during percutaneous coronary intervention (PCI) also results in immediate S1P increase in the coronary sinus that is propagated toward the periphery and correlates with plasma troponin T (Egom et al., 2013). These studies imply myocardial injury as a source of acute S1P release. In contrast, lower plasma S1P concentrations have also been reported in AMI (Knapp et al., 2013; Polzin et al., 2013) and modulation of S1P release from platelets has been suggested to play a role there (Polzin et al., 2013) although HDL-S1P has not been determined. Among patients with similar AMI severity, those that develop a stronger inflammatory response also feature diminished HDL-S1P and a lower propensity of their HDL to activate eNOS (Gomaraschi et al., 2013). In patients with diabetes mellitus, HDL-S1P decreases with increasing HbA1c (Tong et al., 2013), and HDL-S1P is reduced by non-enzymatical glycation (Tong et al., 2014). This is in apparent contradiction to increased HDL-S1P in diabetic patients (Tong et al., 2013) but could be explained by the performed normalization to the lower plasma HDL-C of these patients that is common in diabetes and due to the prevalence of small low-cholesterol HDL particles (Syvanne et al., 1995). In patients with chronic kidney disease and uremic syndrome, HDL-S1P (Prufer et al., 2014) and HDL-apoM (Holzer et al., 2011) are also reduced.

Comparisons among absolute HDL-S1P concentrations in different studies should be carried out with caution for several reasons: (1) as serum contains higher S1P concentrations than plasma HDL isolated from serum (Jing et al., 2015) has higher S1P levels than that isolated from plasma (Sattler et al., 2010, 2015); (2) HDL-S1P has been often normalized to very different HDL components in different studies such as total HDL protein or apolipoprotein AI; it has been also expressed per mol HDL or even per ml HDL isolate (Kontush et al., 2007; Tong et al., 2013; Jing et al., 2015; Sattler et al., 2015); (3) although different methodology of S1P measurement such as LC-MS (Sattler et al., 2015), commercial ELISA kits (Gomaraschi et al., 2013) and HPLC (Kontush et al., 2007; Sattler et al., 2010) should be comparable, they have never been validated side by side in individual studies; (4) there is considerable variability of HDL-S1P among HDL subclasses and, accordingly, their different representation in a certain collective must be considered, and (5) there is still little known about confounders such as, e.g., the impact of medication on HDL-S1P.

## Consequences of Low HDL-S1P for HDL Function and Potential Strategies to Correct HDL Dysfunction by Increasing HDL-S1P

Given the coincidence of low HDL-S1P with HDL dysfunction in human diseases it is imminent to unravel the molecular reasons and pursue potential consequences for HDL function. Conversely, the question should also be addressed whether HDL dysfunction can be linked to diminished HDL-S1P. If so, therapeutic options may be considered to either reverse the biochemical and metabolic changes in HDL resulting in insufficient S1P content or, as an alternative approach, find ways to overcome them by pharmacologically increasing HDL-S1P (Figure 1). Indeed, in CAD and AMI patients, reduced HDL-S1P has been made responsible for the inferior ability of their HDL to activate intracellular signaling including eNOS activation (Gomaraschi et al., 2013; Sattler et al., 2015). In addition, HDL oxidation *in vitro* that renders modifications similar to those observed in CAD (Kontush and Chapman, 2006; Huang et al., 2014) results in diminished HDL-S1P (Sattler et al., 2015). However, understanding the biochemical and molecular determinants of low HDL-S1P in disease may prove particularly difficult if the aim was to prevent or reverse them to restore physiological HDL-S1P levels. A more pragmatic approach would be to try and reinstate normal HDL-S1P content in diseased HDL (provided the HDL alterations causing its deficiency did not preclude it). Actually, a few studies have taken this path and have raised S1P-HDL to try and improve HDL function. Early studies in the mouse heart have shown that administration of human HDL confers cardioprotection against reperfusion injury through HDL-S1P (Theilmeier et al., 2006). Consecutive studies using reconstituted HDL have shown that only HDL manufactured to incorporate S1P are cardioprotective (Brulhart-Meynet et al., 2015). To increase regular HDL-S1P levels, human HDL have been incubated with sphingosine-loaded erythrocytes as physiological S1P donors or with purified S1P and were found to take up large amounts of S1P exceeding those carried within by 10–20-fold (Bode et al., 2010; Sattler et al., 2015). Not only purified but also HDL residing inherently in human plasma effectively acquires S1P from erythrocytes, and C17-S1P injected intravenously in mice in the form of C17-S1P-loaded erythrocytes relocates immediately and almost completely to HDL (Sattler et al., 2015). Similar observations have been made in humans where erythrocyte transfusion resulted in increased plasma and HDL-S1P (Selim et al., 2011). Such pharmacological increases in HDL-S1P have a clear impact on HDL function as S1P-loaded HDL showed enhanced activation of intracellular signaling pathways including eNOS activation in human endothelial cells and a superior vasodilation in isolated arteries (Sattler et al., 2015). Interestingly, not only healthy but also oxidized and glycated HDL, respectively, take up S1P (Tong et al., 2014; Sattler et al., 2015). Although not as efficiently as healthy HDL, oxidized HDL still take up considerable amounts of S1P thereby reaching even higher S1P content than that of healthy HDL (Sattler et al., 2015). This occurs independently of apoM as apoM was almost undetectable in oxidized HDL despite considerable S1P uptake, and murine apoM-deficient HDL was unimpaired in taking up S1P (Sattler et al., 2015). Remarkably, HDL isolated from patients with CAD take up S1P as efficiently and to the same extent as healthy HDL (Sattler et al., 2015). Most importantly, however, S1P supplementation is accompanied by the complete restoration of previously impaired functions of CAD-HDL: S1P-loaded CAD-HDL were as efficient as healthy HDL in activating eNOS and mediating vasorelaxation (Sattler et al., 2015). Similarly, S1P-supplemented glycated HDL re-obtain their ability to induce cyclooxygenase-2 (Tong et al., 2014).

Administration of reconstituted HDL, HDL mimetics and apolipoprotein A-I peptides has been one of the many approaches to raise HDL in attempt to treat cardiovascular disease (Kingwell et al., 2014). Several potentially beneficial effects have been observed in experimental models of disease and encouraging results reported for some of these preparations, respectively, e.g., in acute coronary syndrome where atherosclerotic plaque volume was reduced and inflammation ameliorated (Reddy et al., 2014; Remaley et al., 2014; White et al., 2014). Interestingly, among the functional effects caused by HDL administration are several that have been attributed at least in part to HDL-S1P such as arterial vasodilation. Indeed, impaired flow-mediated vasodilation (FMD) in CAD is associated with low HDL-C (Li et al., 2000) and can be substantially improved by the administration of reconstituted HDL (Spieker et al., 2002). Considering the low HDL-S1P in CAD and the extremely efficient ability of HDL to take up S1P, it is plausible to assume that the acute raise of plasma HDL has lead to an increase in the overall pool of HDL-S1P which could have promoted the S1P-conveyed part of HDL-mediated FMD. Such observations may extend to any of the functionally overlapping effects of administered HDL and those known for HDL-S1P. Accordingly, provided that such indirect increase in HDL-S1P following HDL administration contributes to the observed effects, a more direct intervention aimed at specifically loading S1P onto HDL prior to administration may prove additionally helpful in tackling HDL dysfunction.

### Future Questions

High-density lipoproteins come in a variety of subclasses and particle sizes and are subject to constant dynamic remodeling through their interaction with plasma constituents, tissues and cells. At the same time, HDL are subject to a plethora of pathophysiological modifications in cardiovascular and metabolic diseases. There is sufficient evidence that many of these processes are bound to have an impact on the amounts of S1P that HDL are exposed to, accumulate and transport, and thus influence HDL-S1P-mediated functions. Understanding the molecular events that determine these processes inside and outside of HDL is of major importance. It is also imminent to comprehend how exactly HDL acquire S1P from their surroundings and present it to S1P receptors for signaling. The existence of HDL receptors makes it plausible that signaling by HDL-associated S1P might be prevalent at sites where HDL engages its own receptors and would thus distinguish such targeted S1P signaling from the ubiquitous one ensuing from S1P in its albumin-bound form. In summary, understanding the exact regulation and contribution of HDL-S1P to key HDL functions would allow the design of strategies that employ S1P to enhance the effectiveness of HDL-based therapies.

### Conflict of Interest Statement

The author declares that the research was conducted in the absence of any commercial or financial relationships that could be construed as a potential conflict of interest.
